# Characteristics of Infective Endocarditis in Intravenous Drug Users vs. Non-Users: A Retrospective Study Conducted in Bucharest, Romania

**DOI:** 10.3390/medicina61101785

**Published:** 2025-10-02

**Authors:** Adina-Alexandra Nanu, Dragos Ștefan Lazăr, Corneliu Petru Popescu, Miruna-Ioana Lazăr, Maria Nica, Simin Aysel Florescu

**Affiliations:** 1 Department of Infectious Diseases, “Carol Davila” University of Medicine and Pharmacy, 020021 Bucharest, Romania; adina.nanu@drd.umfcd.ro (A.-A.N.); corneliu.popescu@umfcd.ro (C.P.P.); simin.florescu@umfcd.ro (S.A.F.); 2 “Dr. Victor Babeș” Clinical Hospital of Infectious and Tropical Diseases, 030303 Bucharest, Romania; 3 “Nicolae Malaxa” Clinical Hospital, 022441 Bucharest, Romania

**Keywords:** infective endocarditis, intravenous drug use, PWID, *Staphylococcus aureus*, echocardiography, embolic complications, Romania, long-term mortality, HIV, HCV

## Abstract

*Background and Objectives*: Infective endocarditis (IE) remains a severe infection with high morbidity and mortality, particularly among people who inject drugs (PWID). Data from Eastern Europe are limited, despite the increasing burden of intravenous drug use in the region. *Materials and Methods*: We conducted a retrospective, observational cohort study of 153 patients diagnosed with IE and admitted to the “Dr. Victor Babeș” Clinical Hospital for Infectious and Tropical Diseases in Bucharest, Romania, between August 2019 and July 2024. Patients were classified into PWID (*n* = 51) and non-PWID (*n* = 102). Clinical characteristics, microbiological profiles, echocardiographic findings, complications, and outcomes (in-hospital, 10-week, and 12-month mortality) were compared between groups. *Results*: PWID were significantly younger (mean 34.0 ± 6.6 years vs. 64.3 ± 13.1 years; *p* < 0.001), predominantly male (86.3% vs. 62.7%; *p* = 0.003) and had higher rates of HIV (64.7%) and HCV (98.1%). Right-sided IE and larger vegetations were more common in PWID, whereas non-PWID had more left-sided disease, pre-existing valvular pathology, and prosthetic valve involvement. *Staphylococcus aureus* predominated in PWID (68.6% vs. 27.5%; *p* < 0.001), while non-PWID had more Streptococcus spp. and Coxiella burnetii cases. Embolic complications, particularly pulmonary emboli, and valvular rupture were significantly more frequent in PWID, while non-PWID had higher rates of heart failure and surgical interventions. In-hospital mortality was similar (17.6% vs. 11.8%; *p* = 0.318), but 12-month mortality was higher in PWID (27.5% vs. 13.7%; *p* = 0.038). *Conclusions*: IE in PWID shows a distinct clinical and microbiological profile, with more aggressive complications and worse long-term survival. Tailored management, early diagnosis, harm reduction programs, and dedicated follow-up are urgently needed in this high-risk population.

## 1. Introduction

Infective endocarditis (IE) is a serious and potentially life-threatening condition characterized by infection of the endocardial surface of the heart, typically involving the heart valves. It has a reported mortality rate of up to 30% [1,2,3,4], and the well-known risk factors include pre-existing valvular heart disease, prosthetic heart valves, intravenous drug use, and invasive medical procedures.

People who inject drugs (PWID) have a 50- to 100-fold higher incidence of IE compared to the general population [5,6]. Addressing drug use in this group remains a major challenge due to the complex interplay of social, economic, and psychological factors that are difficult to mitigate. According to the European Union Drugs Agency, injectable drug use continues to decline overall in Europe but remains disproportionately associated with severe health risks, including overdoses and both chronic and acute infectious diseases. Data from the European Syringe Collection & Analysis Project (ESCAPE) reveal a significant diversification: alongside heroin, syringes often contain traces of amphetamines, cocaine, synthetic cathinones, and opioid agonists, frequently in hazardous combinations (euda.europa.eu).

In the context of the ongoing opioid crisis in the United States, the situation in Europe remains uncertain and potentially volatile [7], with a foreseeable risk of increases in the population of individuals who inject drugs, and, consequently, a rise in addiction-related disorders.

Despite the availability of extensive Western and Northern European data, evidence from Eastern Europe—and Romania in particular—remains scarce. A recent Romanian study from Iași (2019–2024) described clinical and etiological aspects of IE, reporting that *Staphylococcus aureus* accounted for 33% of cases and in-hospital mortality reached 5%. However, this study did not stratify outcomes based on injection drug use, as none of the patients in that cohort were PWID [8].

Bucharest, the capital of Romania, is home to the “Victor Babeș” Clinical Hospital for Infectious and Tropical Diseases (VBH), a tertiary-care hospital, one of only two specialized infectious diseases hospitals in the city, serving approximately half of the metropolitan area and the southeastern regions of the country. According to the Romanian National Anti-Drug Agency, the highest prevalence of intravenous drug use in Romania is recorded in Bucharest and its surrounding areas [9].

In response to the limited regional data on infective endocarditis among PWID, we conducted a retrospective study including 153 IE cases at VBH in Bucharest from 2019 to 2024. We compared clinical characteristics, microbiological profiles, echocardiographic findings, and short- and long-term mortality (30-day, 10-week, and 12-month) between PWID and non-PWID groups. The findings are intended to inform context-specific clinical practices and improve regional prevention efforts.

## 2. Materials and Methods

### 2.1. Study Design and Inclusion Criteria

This observational, single-center cohort study was carried out at VBH in Bucharest, Romania and included adult patients (≥18 years old) hospitalized between August 2019 and July 2024 with a definite or possible diagnosis of infective endocarditis (IE), who provided informed consent regarding data use for research purposes.

According to recent National Anti-Drug Agency reports, Bucharest and nearby areas face the highest rates of intravenous drug use in Romania.

### 2.2. Exclusion Criteria

Patients under 18 years old, those with incomplete clinical or paraclinical records, and those who did not consent to data use for research were excluded. A total of 153 patients met the inclusion criteria. Based on personal history, medical records, or referral from addiction services, 51 were classified as people who inject drugs (PWID) and 102 as non-PWID.

### 2.3. Admission and Diagnostic Procedures

At admission, patients were evaluated for signs of systemic infection. Clinical suspicion of IE was based on medical and behavioral history, symptom type and duration, laboratory abnormalities, and imaging when indicated (chest X-ray, abdominal/pelvic ultrasound, or PET scan). In line with hospital protocols, three sets of blood cultures (one aerobic and one anaerobic bottle per set) were collected at least one hour apart, preferably before the initiation of empiric intravenous antibiotics.

Diagnosis was established according to the Duke criteria valid at the time of hospitalization. Patients admitted before 2023 were evaluated using the original Duke criteria, while those admitted in 2023 or later were assessed using the updated Duke-ISCVID criteria.

For patients with persistently negative cultures, additional work-up for culture-negative IE was performed. When clinically appropriate, serological tests for *Coxiella burnetii* and *Bartonella* spp. were obtained, following local protocol—an approach already in place before the updated Duke criteria were introduced.

To ensure diagnostic consistency across the study period, all included cases were retrospectively reclassified using the updated Duke criteria and the MEDcalc online calculator. Cases were categorized as definite, possible, or rejected IE. Only definite and possible IE cases were included in the final analysis, since both received the same clinical management.

### 2.4. Data Retrieval

Data were extracted from medical records and discharge summaries, includingage, sex, body-mass index (BMI), housing status, intravenous drug use, tobacco, alcohol;

Chronic infections such asHIV, HBV, HCV, syphilis, signs, symptoms, duration, disease progression.

Paraclinical findings: laboratory data (inflammatory markers, pathogen-specific serology), echocardiography (vegetation size, valve involvement, valve rupture), and imaging for embolic events.

All data were anonymized before analysis, in line with institutional ethical standards and the Declaration of Helsinki. The study protocol was approved by the hospital’s ethics committee (approval number 14958/11 September2019).

### 2.5. Outcomes

The primary outcome was all-cause mortality one year after IE diagnosis. Secondary outcomes included complications, need for cardiovascular surgery, and length of hospital stay.

### 2.6. Statistical Analysis

Descriptive statistics (means, medians, standard deviations, interquartile ranges) were used for quantitative data; categorical variables were reported as frequencies and percentages. Group comparisons were performed using Pearson’s chi-square or Fisher’s exact test for categorical variables and Mann–Whitney U test for continuous variables. Normality of distribution was tested with Kolmogorov–Smirnov and Shapiro–Wilk, and visually confirmed with histograms and Q-Q plots. All statistical analyses were conducted in SPSS v26.0 (IBM, Chicago, IL, USA) and GraphPad Prism v8.4.2, which were also used for figure creation. A *p*-value < 0.05 was considered statistically significant, with a 95% confidence interval.

## 3. Results

### 3.1. Demographic and Clinical Characteristics

A total of 153 patients diagnosed with IE were included: 51 (33.3%) were people who inject drugs (PWID), while the remaining 102 (66.7%) were non-PWID (Table 1).

The PWID group was significantly younger (mean 34.0 ± 6.6 years) compared to non-PWID (64.3 ± 13.1 years, *p* < 0.001). Most PWID were men (86.3% vs. 62.7%; *p* = 0.003) and were less likely to have stable housing (60.8% vs. 98%; *p* < 0.001).

Body mass index (BMI) was lower among PWID (19.2 ± 2.4 kg/m^2^ vs. 26.7 ± 5.9 kg/m^2^; *p* < 0.001). HIV (64.7%) and HCV (98.1%) were highly prevalent in PWID (*p* < 0.001 for both), while HBV and syphilis showed no significant differences. All PWID were smokers, and alcohol consumption was also more frequent (86.3% vs. 26.5%; *p* < 0.001). A history of previous IE was similar between groups (15.7% vs. 12.7%; *p* = 0.618).

### 3.2. Valvular Involvement

Underlying heart valve disease (Table 2) was more common in non-PWID (60.4% vs. 3.9%; *p* < 0.001). Prosthetic valve IE was also higher in non-PWID (24.5% vs. 2%; *p* = 0.002). Most patients met Duke criteria for definite IE (90.2% vs. 89%; *p* = 0.821). The mean number of major Duke criteria was the same, but PWID had more minor criteria (3.3 vs. 2.3; *p* < 0.001).

### 3.3. Clinical Presentation

PWID presented more frequently with fever (96.1% vs. 70.6%; *p* < 0.001), chills (60.8% vs. 39.2%; *p* = 0.012), cough (72.5% vs. 16.7%; *p* < 0.001), and myalgia (29.4% vs. 8.8%; *p* = 0.001). Cardiopulmonary signs such as tachycardia, higher respiratory rate, and pulmonary crackles were more common in PWID. Lymphadenopathy, abdominal tenderness, and hepatomegaly were also significantly higher. Chest radiographs showed pulmonary consolidation in 70.6% of PWID vs. 24.5% of non-PWID (*p* < 0.001). Heart failure was more frequent among non-PWID (50% vs. 13.7%; *p* < 0.001) (Table 3).

### 3.4. Microbiological Findings

Positive blood cultures were positive in most patients (74.5% non-PWID vs. 78.4% PWID; *p* = 0.593). Staphylococcus spp. were significantly more frequent among PWID (68.6% vs. 27.5%; *p* < 0.001), particularly *S. aureus*. Non-PWID more often had Streptococcus spp. and culture-negative IE, with six confirmed cases of *Coxiella burnetii* (Table 4).

### 3.5. Laboratory Findings

**Figure 1:**
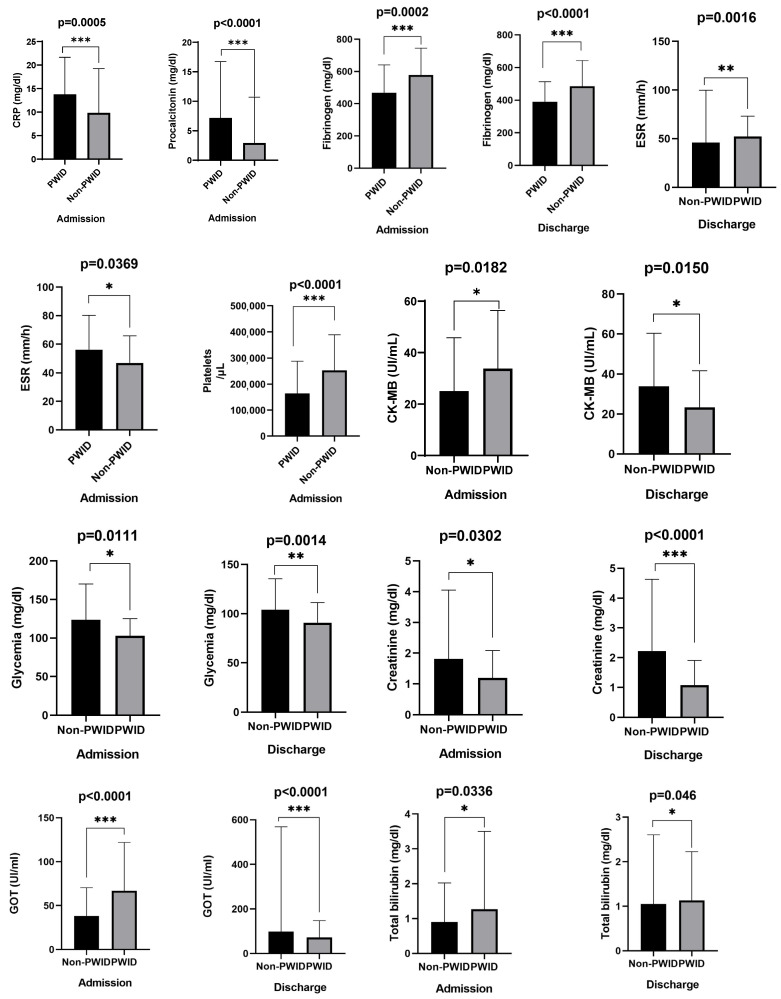
Laboratory findings with statistically significant differences between PWID and non-PWID. * (*p* < 0.05), ** (*p* < 0.01), *** (*p* < 0.001).

PWID showed stronger inflammatory responses at admission: higher CRP, procalcitonin, and ESR. Fibrinogen was higher in non-PWID. Platelet counts were significantly lower in PWID at admission but recovered by discharge. Liver enzymes (AST) and total bilirubin were higher in PWID, while creatinine and glucose were higher in non-PWID (Table 5, Figure 1).

### 3.6. Complications and Outcomes

According to Table 6, PWID had more embolic complications (60.8% vs. 34.3%; *p* = 0.002), especially pulmonary embolism (83.8% vs. 17.1%; *p* < 0.0001). Valve rupture was also more common in PWID (27.5% vs. 5.9%; *p* < 0.001).

Non-PWID more often developed congestive heart failure (50% vs. 13.7%; *p* < 0.001) and were scheduled for cardiac surgery (15.7% vs. 3.9%; *p* = 0.033).

Mortality rates were similar in hospital and at 10 weeks, but 12-month mortality was higher in PWID (27.5% vs. 13.7%; *p* = 0.038). Discharge against medical advice was more frequent in PWID (35.3% vs. 6.9%; *p* < 0.001).

**Figure 2:**
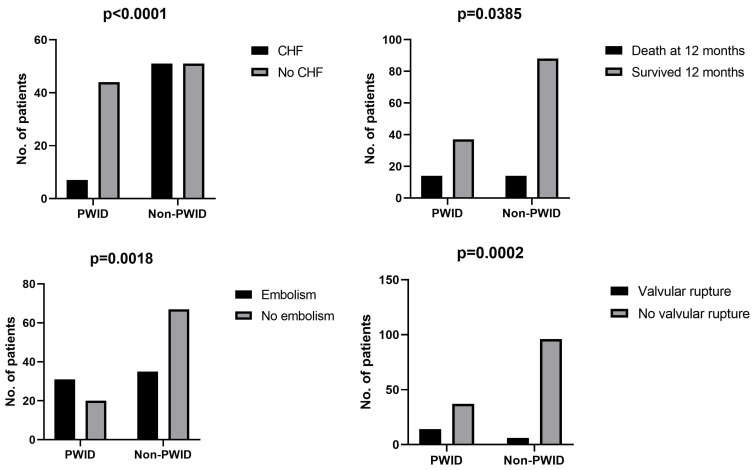
Statistically significant differences between PWID and non-PWID patients regarding clinical evolution.

A summary of the main differences between the two groups is provided in Table 7 and Figure 2.

## 4. Discussion

We retrospectively analyzed infective endocarditis presentation in people who inject drugs vs. non-PWID that were admitted to an infectious diseases hospital in Bucharest, Romania, between 2019 and 2024. The findings reveal deep divergences between the two groups—not just in age and social background, but also in how the disease manifests, the bacteria involved, and what happens to patients over time.

As anticipated, PWID were younger, predominantly of male sex, experienced frequently unstable life situations, and exhibited other risk behaviors such as smoking and alcohol consumption [10,11,12,13]. The prevalence of HIV (64.7%) and HCV (98.1%) was alarmingly high in this group, surpassing that reported in some Western European studies [14,15,16]. This probably mirrors disparities in access to harm reduction services.

On a cardiac level, PWID were more prone to have right-sided IE, affecting the tricuspid valve, compared to other patients, with larger vegetation. On the other hand, non-PWID had more left-sided disease, frequently on a prior damaged or prosthetic valve.

Microbiology held a consistent narrative—*Staphylococcus aureus* was the main culprit among PWID, and frighteningly many of these were MRSA, adding further complexity to our initial treatment options [6,7]. Non-PWID not infrequently had infections due to *Streptococcus* species or culture-negative IE, sometimes linked to *Coxiella burnetii*. This aligns with previous reports suggesting that *Coxiella burnetii* infections—beyond endocarditis—are starting to become relatively common in Romania [17,18].

Clinically, PWID showed more severe systemic inflammation, reflected by higher CRP, procalcitonin, and ESR values at admission. Despite significant clinical manifestations (fever, chills, tachycardia, respiratory signs), the frequency of heart failure was considerably lower among PWID, likely due to the absence of underlying structural heart disease. This aligns with other studies indicating different disease mechanisms between the two groups [14,19].

Regarding complications, PWID had significantly more embolic events, particularly pulmonary emboli, and a higher rate of valvular rupture, suggesting a more aggressive disease course. In contrast, non-PWID experienced more frequent heart failure and surgical interventions, findings consistent with valve pathology patterns [20,21]. It is also worth noting that in the cases of many PWID, surgery is often postponed or not provided at all unless the patient and family show they are addressing ongoing drug use. And because the risk remains, surgeons might choose to delay or avoid operating if it is not absolutely critical.

Mortality patterns were particularly telling. Although in-hospital and short-term mortality did not differ significantly between groups, 12-month mortality was twice as high in PWID (27.5% vs. 13.7%), in agreement with existing evidence indicating poor long-term outcomes in this population [15,16,17,18,22]. Discharge against medical advice was also much more common among PWID, which may contribute to treatment non-completion and subsequent adverse outcomes.

The study has strengths in having a relatively large number of cases for a single center, meticulous reclassification according to the updated Duke criteria, and detailed clinical and microbiological data. But there are limitations: the retrospective design, the absence of long-term follow-up beyond one year, and potential selection bias because it was performed in a referral center.

In conclusion, our findings emphasize the distinct profile of IE in PWID, characterized by right-sided disease, *Staphylococcus aureus* predominance, more frequent embolic complications, and worse long-term survival. These data underline the need for tailored management strategies, including early diagnosis, harm reduction interventions, and post-discharge follow-up programs specifically targeting PWID. Regional and national strategies should also address the broader public health challenges posed by intravenous drug use, particularly in high-risk urban settings such as Bucharest.

## 5. Conclusions

This study underscores distinctions between those who inject drugs and the remaining patient population that develops infective endocarditis.

PWID are typically younger, frequently exhibit involvement of the right side of the heart, and develop complications such as septic pulmonary emboli and rapid valvular destruction. Although the disease is typically aggressive, most of these patients are not accepted for surgery unless they demonstrate a strong commitment to dealing with their drug dependence, which is still a major barrier to care.

On the other hand, non-PWID are typically older, have increased left-sided valve involvement, with heart failure occurring more often and have higher operative rates.

These findings are a reminder of what it means to treat infective endocarditis: it is not always just about treating an infection; it is sometimes about grappling with the social context of each patient. For PWID, medical treatment needs to be combined with addiction treatment, counseling, and family involvement. Even the best therapy may not improve long-term outcomes if the underlying problems are not addressed.

## Figures and Tables

**Table 1:** Demographic characteristics of patients.

	Non-PWID (*n* = 102)	PWID (*n* = 51)	*p*-Value
Female sex, *n* (%)	38 (37.3)	7 (13.7)	0.003
Male sex, *n* (%)	64 (62.7)	44 (86.3)	
Age, years (mean ± SD; median [IQR])	64.29 ± 13.12; 67 [56–73.25]	34.04 ± 6.57; 34 [29–40]	<0.001
BMI (kg/m^2^) (mean ± SD; median [IQR])	26.68 ± 5.91; 25.8 [22.5–31]	19.19 ± 2.36; 18.6 [17.8–20]	<0.001
Stable housing, *n* (%)	100 (98.0)	31 (60.8)	<0.001
Smoking, *n* (%)	39 (38.2)	51 (100.0)	<0.001
Alcohol consumption, *n* (%)	27 (26.5)	44 (86.3)	<0.001
Non-infectious comorbidities, *n* (%)	101 (99.0)	11 (21.6)	<0.001
Infectious comorbidities, *n* (%)	66 (64.7)	37 (72.5)	0.330

Abbreviations: PWID = people who inject drugs; BMI = body mass index; SD = standard deviation; IQR = interquartile range.

**Table 2:** Relevant history and valvular involvement in patients with infective endocarditis.

	Non-PWID (*n* = 102)	PWID (*n* = 51)	*p*-Value
Prosthetic valve IE, *n* (%)	25 (24.5)	1 (2.0)	0.002
Biological valve, *n* (%)	12 (48.0)	1 (100.0)	0.061
Mechanical valve, *n* (%)	12 (48.0)	0 (0.0)	0.009
Pacemaker-related IE, *n* (%)	1 (4.0)	0 (0.0)	1.000
Pre-existing valve disease, *n* (%)	61 (60.4)	2 (3.9)	<0.001
Mitral valve disease, *n* (%)	40 (65.6)	1 (50.0)	0.649
Tricuspid valve disease, *n* (%)	9 (8.8)	0 (0.0)	0.030
Aortic valve disease, *n* (%)	46 (45.1)	0 (0.0)	<0.001
Valvular stenosis, *n* (%)	14 (13.7)	0 (0.0)	0.005
Valvular insufficiency, *n* (%)	37 (36.3)	1 (2.0)	<0.001
Both stenosis and insufficiency, *n* (%)	7 (6.9)	0 (0.0)	0.096
Pulmonary valve disease, *n* (%)	2 (2.0)	0 (0.0)	1.000
Vegetation identified, *n* (%)	91 (89.2)	47 (92.2)	0.564
TTE performed, *n* (%)	64 (62.7)	47 (92.2)	<0.001
TEE performed, *n* (%)	44 (43.1)	3 (5.9)	<0.001
PET-CT performed, *n* (%)	1 (1.0)	0 (0.0)	1.000
IE affecting tricuspid valve, *n* (%)	8 (7.8)	37 (72.5)	<0.001
IE affecting mitral valve, *n* (%)	53 (52.0)	11 (21.6)	<0.001
IE affecting aortic valve, *n* (%)	48 (47.1)	7 (13.7)	<0.001
IE affecting pulmonary valve, *n* (%)	0 (0.0)	1 (2.0)	0.336
IE affecting interventricular septum, *n* (%)	0 (0.0)	1 (2.0)	0.336
Vegetation size, longitudinal (cm, mean ± SD; median [IQR])	1.06 ± 1.22; 0.8 [0.5–1.25]	1.76 ± 0.84; 1.5 [1.2–2.1]	<0.001
Vegetation size, transverse (cm, mean ± SD; median [IQR])	0.72 ± 0.46; 0.5 [0.5–0.96]	1.20 ± 0.58; 1.0 [0.8–1.7]	<0.001
More than one valve affected, *n* (%)	4 (3.9)	4 (7.8)	0.442
Major Duke criteria (mean ± SD; median [IQR])	1.68 ± 0.47; 2 [1–2]	1.68 ± 0.47; 2 [1–2]	0.894
Minor Duke criteria (mean ± SD; median [IQR])	2.3 ± 0.8; 2 [2–3]	3.32 ± 0.62; 3 [3–4]	<0.001

Abbreviations: IE = infective endocarditis; PWID = people who inject drugs; TTE = transthoracic echocardiography; TEE = transesophageal echocardiography; PET-CT = positron emission tomography–computed tomography; SD = standard deviation; IQR = interquartile range.

**Table 3:** Clinical and paraclinical findings at admission.

	Non-PWID (*n* = 102)	PWID (*n* = 51)	*p*-Value
Subfebrility, *n* (%)	7 (6.9)	0 (0.0)	0.096
Fever, *n* (%)	72 (70.6)	49 (96.1)	<0.001
Chills, *n* (%)	40 (39.2)	31 (60.8)	0.012
Maximum temperature (°C), mean ± SD, median [IQR]	38.14 ± 1.08, 38.3 [37.8–38.7]	38.78 ± 0.71, 38.7 [38.2–39.2]	<0.001
Intense sweating, *n* (%)	18 (17.6)	16 (31.4)	0.054
Dyspnea, *n* (%)	25 (24.5)	19 (37.3)	0.101
Palpitations, *n* (%)	11 (10.8)	2 (3.9)	0.221
Cough, *n* (%)	17 (16.7)	37 (72.5)	<0.001
Edema, *n* (%)	20 (19.6)	13 (25.5)	0.404
Precordial pain, *n* (%)	9 (8.8)	4 (7.8)	1.000
Asthenia, *n* (%)	96 (94.1)	47 (92.2)	0.732
Myalgias, *n* (%)	9 (8.8)	15 (29.4)	0.001
Arthralgias, *n* (%)	8 (7.8)	7 (13.7)	0.249
Fatigability, *n* (%)	96 (94.1)	49 (96.1)	0.719
Inappetence, *n* (%)	95 (93.1)	45 (88.2)	0.360
Chest pain, *n* (%)	6 (5.9)	6 (11.8)	0.216
Syncope, *n* (%)	4 (3.9)	7 (13.7)	0.043
Systolic BP ≤ 90 mmHg, *n* (%)	7 (6.9)	8 (15.7)	0.084
Heart rate (bpm), mean ± SD, median [IQR]	91.02 ± 17.26, 88.5 [80–101.2]	101.18 ± 17.57, 100 [90–113]	<0.001
Respiratory rate, mean ± SD, median [IQR]	19.52 ± 3.79, 19 [17–25]	22.84 ± 6.91, 20 [18–25]	0.005
SpO_2_ (%), mean ± SD, median [IQR]	95.81 ± 3.4, 97 [95–98]	94.92 ± 4.4, 97 [94–98]	0.263
Pathological lung sounds, *n* (%)	28 (27.5)	25 (49.0)	0.008
Crepitants, *n* (%)	23 (82.1)	8 (32.0)	0.0003
Bronchial rales, *n* (%)	5 (17.8)	17 (68.0)	—
Systolic murmur, *n* (%)	63 (61.8)	33 (64.7)	0.723
Diastolic murmur, *n* (%)	4 (3.9)	2 (3.9)	1.000
Palpable lymph nodes, *n* (%)	3 (2.9)	32 (62.7)	<0.001
Abdominal tenderness, *n* (%)	8 (7.9)	19 (37.3)	<0.001
Hepatomegaly, *n* (%)	12 (11.8)	37 (72.5)	<0.001
Diarrhea, *n* (%)	8 (7.8)	7 (13.7)	0.249
Focal neurological signs, *n* (%)	24 (23.5)	1 (2.0)	0.001
Meningitis signs, *n* (%)	8 (7.8)	4 (7.8)	1.000
Alveolar lung disease on X-ray, *n* (%)	25 (24.5)	36 (70.6)	<0.001
Pleural fluid on X-ray, *n* (%)	26 (25.5)	15 (29.4)	0.606

Abbreviations: PWID = people who inject drugs; BP = blood pressure; bpm = beats per minute; SpO_2_ = peripheral capillary oxygen saturation; IQR = interquartile range.

**Table 4:** Microbiological findings in patients with infective endocarditis.

	Non-PWID (*n* = 102)	PWID (*n* = 51)	*p*-Value
Identified microorganism, *n* (%)	76 (74.5)	40 (78.4)	0.593
Staphylococcus spp., *n* (%)	28 (27.5)	35 (68.6)	<0.001
Methicillin-resistant *S. aureus* (MRSA), *n* (%)	9 (8.8)	14 (27.5)	0.002
Methicillin-sensitive *S. aureus* (MSSA), *n* (%)	11 (10.9)	21 (41.2)	<0.001
Streptococcus spp. (*pyogenes*, *agalactiae*, *dysgalactiae*, *gallolyticus*, *anginosus*, *constellatus*, *gordonii*, *mitis*, *oralis*, *salivarius*, *sanguinis*, *viridans*), *n* (%)	23 (22.5)	6 (11.8)	0.109
*Coxiella burnetii*, *n* (%)	6 (5.9)	0 (0.0)	0.179
*Klebsiella* spp., *n* (%)	3 (3.0)	0 (0.0)	0.551
*Enterococcus* spp., *n* (%)	11 (10.8)	1 (2.0)	0.062
Coexisting fungal infection, *n* (%)	5 (4.9)	0 (0.0)	0.170
Polymicrobial acute infection, *n* (%)	11 (10.8)	2 (3.9)	0.221

Abbreviations: IE = infective endocarditis; PWID = people who inject drugs; MRSA = methicillin-resistant *Staphylococcus aureus*; MSSA = methicillin-sensitive *Staphylococcus aureus*.

**Table 5:** Laboratory data at admission and discharge.

	Non-PWID (*n* = 102)	PWID (*n* = 51)	*p*-Value
Leukocytes (×10^3^/µL)			
Admission, mean ± SD, median [IQR]	11.61 ± 4.99, 10.9 [7.7–14.3]	12.91 ± 6.26, 12.3 [9.2–16.6]	0.199
Discharge, mean ± SD, median [IQR]	8.89 ± 4.19, 8.1 [5.9–10.7]	12.59 ± 10.97, 9.75 [5.8–15.3]	0.092
C-Reactive Protein (mg/dL)			
Admission	9.81 ± 9.43, 6.88 [2.6–13.5]	13.75 ± 7.9, 13.9 [8.4–18.5]	<0.001
Discharge	4.05 ± 5.7, 1.7 [0.5–5.4]	5.19 ± 5.8, 2.83 [0.6–9.5]	0.286
Procalcitonin (mg/dL)			
Admission	2.90 ± 7.79, 0.29 [0.11–1.45]	7.18 ± 9.54, 2.65 [0.67–10]	<0.001
Discharge	0.72 ± 2.93, 0.11 [0.04–0.5]	2.80 ± 6.95, 0.11 [0.05–1.27]	0.102
Fibrinogen (mg/dL)			
Admission	578.5 ± 165.6, 562 [479–685]	467.1 ± 173.8, 451 [338–589]	<0.001
Discharge	485.8 ± 156.6, 487.5 [390–559]	390.0 ± 123.6, 391.5 [300–466]	<0.001
ESR (mm/h)			
Admission	47.0 ± 18.9, 44.5 [33–55]	56.2 ± 24.0, 55 [38–71]	0.036
Discharge	46.1 ± 53.7, 38 [26–49]	52.3 ± 20.9, 50 [33.8–63.5]	0.002
Platelets (×10^3^/µL)			
Admission	252.8 ± 135.7, 241 [173–325]	164.0 ± 123.9, 120.3 [57–259]	<0.001
Discharge	229.5 ± 115.3, 218.5 [157–275]	232.6 ± 132.7, 212 [124.5–355]	0.900
Hemoglobin (g/dL)			
Admission	10.53 ± 1.77, 10.4 [9.1–11.8]	9.97 ± 1.95, 9.8 [9.0–10.7]	0.081
Discharge	10.42 ± 1.79, 10.1 [8.8–11.8]	10.16 ± 1.84, 9.9 [8.9–11.1]	0.547
Prothrombin Index (%)			
Admission	71.3 ± 20.0, 74 [59.8–85]	72.5 ± 14.0, 75 [64–81]	0.895
Discharge	72.1 ± 23.6, 77 [56–91]	81.7 ± 17.0, 86.5 [73.8–93.5]	0.035
D-dimers (mg/L)			
Admission	3.42 ± 3.35, 2.36 [1.4–4.4]	3.60 ± 2.55, 3.5 [1.3–5.5]	0.337
Discharge	2.88 ± 3.24, 2.06 [1.2–3.1]	2.81 ± 2.03, 2.42 [1.3–3.5]	0.440
Troponin I ultrasensitive (ng/mL)			
Admission	162.2 ± 375.9, 30 [11–56.9]	155.0 ± 293.7, 55 [21.5–155]	0.057
Discharge	268.5 ± 977.8, 28.95 [11–44.5]	139.3 ± 488.7, 32.5 [16–93.3]	0.393
CK-MB (U/L)			
Admission	25.1 ± 20.7, 19.3 [10.2–33]	33.8 ± 22.6, 26.3 [16.3–50.6]	0.019
Discharge	23.3 ± 18.3, 19 [12.6–23]	33.9 ± 26.5, 27 [16.2–43]	0.015
Potassium (mEq/L)			
Admission	4.03 ± 0.7, 4.1 [3.5–4.5]	3.8 ± 0.7, 3.7 [3.3–4.1]	0.015
Discharge	4.24 ± 0.8, 4.2 [3.8–4.7]	4.11 ± 0.7, 4.05 [3.8–4.6]	0.150
Sodium (mEq/L)			
Admission	136.9 ± 4.7, 137 [134.5–140.3]	133.3 ± 6.0, 134 [129–138]	<0.001
Discharge	137.6 ± 4.8, 138 [135–140.3]	133.3 ± 6.0, 134 [134–139]	0.255
Glycemia (mg/dL)			
Admission	123.6 ± 46.5, 112 [91–135]	102.9 ± 22.2, 100 [88–117]	0.011
Discharge	104.0 ± 31.5, 97.4 [84–117]	90.8 ± 20.6, 87.5 [76–98.3]	0.001
Creatinine (mg/dL)			
Admission	1.81 ± 2.24, 1.1 [0.83–1.47]	1.19 ± 0.89, 1.0 [0.7–1.17]	0.030
Discharge	2.22 ± 2.41, 1.19 [0.89–2.84]	1.08 ± 0.82, 0.86 [0.7–1.12]	<0.001
GPT/ALT (U/L)			
Admission	42.1 ± 35.2, 32 [20.5–50]	46.2 ± 40.4, 30 [21–53]	0.761
Discharge	70.1 ± 187.5, 26.5 [19–60.2]	49.4 ± 38.0, 37 [21–65]	0.144
GOT/AST (U/L)			
Admission	38.2 ± 32.2, 27.5 [19–47]	66.8 ± 55.1, 56 [29–76]	<0.001
Discharge	98.1 ± 470.5, 29 [21–43.3]	72.4 ± 75.0, 51.5 [33.9–86.3]	<0.001
LDH (U/L)			
Admission	287.3 ± 112.8, 268.5 [198.8–367]	373.0 ± 232.1, 306 [232–455]	0.045
Discharge	284.7 ± 102.2, 281 [211–339]	357.6 ± 324.3, 288 [211–388]	0.466
Total bilirubin (mg/dL)			
Admission	0.90 ± 1.12, 0.57 [0.36–1.04]	1.26 ± 2.23, 0.86 [0.54–1.10]	0.034
Discharge	1.05 ± 1.55, 0.7 [0.5–0.9]	1.13 ± 1.09, 0.9 [0.8–1.1]	0.005

Abbreviations: PWID = people who inject drugs; ESR = erythrocyte sedimentation rate; PI = prothrombin index; CK-MB = creatine kinase-MB; GOT/AST = glutamate oxaloacetate transaminase/aspartate aminotransferase; GPT/ALT = glutamate pyruvate transaminase/alanine aminotransferase; LDH = lactate dehydrogenase; IQR = interquartile range.

**Table 6:** Evolutive characteristics and outcomes of patients with infective endocarditis.

	Non-PWID (*n* = 102)	PWID (*n* = 51)	*p*-Value
**In-hospital course**			
Congestive heart failure—*n* (%)	51 (50%)	7 (13.7%)	<0.001
ICU admission—*n* (%)	16 (15.7%)	13 (25.5%)	0.144
Transfer to emergency hospitals—*n* (%)	4 (3.9%)	3 (5.9%)	0.584
Cardiovascular surgery scheduled—*n* (%)	16 (15.7%)	2 (3.9%)	0.033
**Hospital outcomes**			
Fatal cases—*n* (%)	12 (11.8%)	9 (17.6%)	0.318
Hospital discharge on demand—*n* (%)	7 (6.9%)	18 (35.3%)	<0.001
Improved clinical status at discharge—*n* (%)	76 (74.5%)	36 (70.6%)	0.605
Hospitalization days, mean ± SD, median [IQR]	30.7 ± 16.2, 30.5 [18–41.3]	30.4 ± 22.5, 30 [9–44]	0.568
**Mortality**			
At 10 weeks—*n* (%)	13 (12.7%)	10 (19.6%)	0.262
At 12 months—*n* (%)	14 (13.7%)	14 (27.5%)	0.038
**Complications**			
Embolic complications—*n* (%)	35 (34.3%)	31 (60.8%)	0.002
Pulmonary embolism—*n* (%)	6 (17.1%)	26 (83.8%)	<0.0001
Abdominal organ embolism—*n* (%)	11 (31.4%)	4 (12.9%)	0.086
Valvular rupture—*n* (%)	6 (5.9%)	14 (27.5%)	<0.001
IE recurrence after 6 months—*n* (%)	0 (0%)	2 (3.9%)	0.109

Abbreviations: PWID = people who inject drugs; ICU = intensive care unit; IE = infective endocarditis; IQR = interquartile range.

**Table 7:** Summary of key differences between PWID and non-PWID with IE.

Domain	Non-PWID (*n* = 102)	PWID (*n* = 51)	*p*-Value
Demographics	Older age (mean 64 y), balanced sex (63% male), stable housing in 98%	Younger age (mean 34 y), predominantly male (86%), unstable housing in 39%	<0.001
Comorbidities	Frequent non-infectious comorbidities (99%)	Rare non-infectious comorbidities (22%)	<0.001
Infections	HIV absent; HCV rare (2%)	HIV in 65%; HCV in 98%	<0.001
Valve involvement	Mostly left-sided IE (mitral 52%, aortic 47%), prosthetic valve IE 24%	Mostly right-sided IE (tricuspid 73%), larger vegetations	<0.001
Microbiology	Streptococcus spp. 23%; culture-negative (6 cases with Coxiella burnetii)	*Staphylococcus aureus* 68% (MRSA 28%)	<0.001
Clinical presentation	More heart failure (50%); less fever and systemic inflammation	More fever (96%), cough (73%), higher CRP/procalcitonin/ESR	<0.001
Complications	More heart failure; more surgery (16%)	More embolic events (61%), pulmonary embolism (84%), valve rupture (28%)	<0.001
Outcomes	In-hospital mortality 12%; 12-month mortality 14%	In-hospital mortality 18%; 12-month mortality 28%	0.038
Discharge	AMA discharge 7%	AMA discharge 35%	<0.001

Abbreviations: IE—infective endocarditis; PWID—people who inject drugs; AMA—against medical advice; CRP—C-reactive protein; ESR—erythrocyte sedimentation rate; MRSA—methicillin-resistant *Staphylococcus aureus*.

## Data Availability

The data presented in this study are available on request from the corresponding author. The data are not publicly available due to patient confidentiality.

## Abbreviations

The following abbreviations are used in this manuscript:

IE	Infective Endocarditis
PWID	People Who Inject Drugs
HIV	Human Immunodeficiency Virus
HBV	Hepatitis B Virus
HCV	Hepatitis C Virus
CRP	C-Reactive Protein
ESR	Erythrocyte Sedimentation Rate
TTE	Transthoracic Echocardiography
TEE	Transesophageal Echocardiography
PET-CT	Positron Emission Tomography–Computed Tomography
CHF	Congestive Heart Failure
MRSA	Methicillin-Resistant *Staphylococcus aureus*
MSSA	Methicillin-Sensitive *Staphylococcus aureus*
AST (GOT)	Aspartate Aminotransferase
ALT (GPT)	Alanine Aminotransferase
LDH	Lactate Dehydrogenase
ICU	Intensive Care Unit
BMI	Body Mass Index
PI	Prothrombin Index
ESCAPE	European Syringe Collection and Analysis Project
